# The methane-driven interaction network in terrestrial methane hotspots

**DOI:** 10.1186/s40793-022-00409-1

**Published:** 2022-04-05

**Authors:** Thomas Kaupper, Lucas W. Mendes, Anja Poehlein, Daria Frohloff, Stephan Rohrbach, Marcus A. Horn, Adrian Ho

**Affiliations:** 1 Institute for Microbiology, Leibniz Universität Hannover, Herrenhäuser Str. 2, 30419 Hannover, Germany; 2 Center for Nuclear Energy in Agriculture, University of São Paulo CENA-USP, Piracicaba, SP Brazil; 3 Department of Genomic and Applied Microbiology and Göttingen Genomics Laboratory, Institute of Microbiology and Genetics, George-August University Göttingen, Grisebachstr. 8, 37077 Göttingen, Germany

**Keywords:** Stable-isotope probing, Microbial interaction, Aerobic methanotrophs, Freshwater methanotrophs, Methane bio-filter

## Abstract

**Background:**

Biological interaction affects diverse facets of microbial life by modulating the activity, diversity, abundance, and composition of microbial communities. Aerobic methane oxidation is a community function, with emergent community traits arising from the interaction of the methane-oxidizers (methanotrophs) and non-methanotrophs. Yet little is known of the spatial and temporal organization of these interaction networks in naturally-occurring complex communities. We hypothesized that the assembled bacterial community of the interaction network in methane hotspots would converge, driven by high substrate availability that favors specific methanotrophs, and in turn influences the recruitment of non-methanotrophs. These environments would also share more co-occurring than site-specific taxa.

**Results:**

We applied stable isotope probing (SIP) using ^13^C-CH_4_ coupled to a co-occurrence network analysis to probe trophic interactions in widespread methane-emitting environments, and over time. Network analysis revealed predominantly unique co-occurring taxa from different environments, indicating distinctly co-evolved communities more strongly influenced by other parameters than high methane availability. Also, results showed a narrower network topology range over time than between environments. Co-occurrence pattern points to *Chthoniobacter* as a relevant yet-unrecognized interacting partner particularly of the gammaproteobacterial methanotrophs, deserving future attention. In almost all instances, the networks derived from the ^13^C-CH_4_ incubation exhibited a less connected and complex topology than the networks derived from the ^unlabelled^C-CH_4_ incubations, likely attributable to the exclusion of the inactive microbial population and spurious connections; DNA-based networks (without SIP) may thus overestimate the methane-dependent network complexity.

**Conclusion:**

We demonstrated that site-specific environmental parameters more strongly shaped the co-occurrence of bacterial taxa than substrate availability. Given that members of the interactome without the capacity to oxidize methane can exert interaction-induced effects on community function, understanding the co-occurrence pattern of the methane-driven interaction network is key to elucidating community function, which goes beyond relating activity to community composition, abundances, and diversity. More generally, we provide a methodological strategy that substantiates the ecological linkages between potentially interacting microorganisms with broad applications to elucidate the role of microbial interaction in community function.

**Supplementary Information:**

The online version contains supplementary material available at 10.1186/s40793-022-00409-1.

## Background

Microbial interactions are widespread, leading to a plethora of interdependent relationships with stimulatory and inhibitory effects on community function [1–4]. It is becoming evident that aerobic methane oxidation is a community function, whereby microorganisms lacking the enzymatic repertoire to oxidize methane are also relevant. These microorganisms (non-methanotrophs) play a significant role, stimulating methanotrophic activity and growth, and increasing methanotroph–mediated micropollutant degradation [2, 5, 6]. Interestingly, the accompanying non-methanotrophs have also been implicated in the resilience of methanotrophic activity during recovery from disturbances [7–9]. Emergent properties may thus arise from the interaction of the methanotrophs and non-methanotrophs, both constituting the “methanotroph interactome” defined here as the consortium of co-occurring microorganisms that can be tracked via the flow of ^13^C-CH_4_ from the methanotrophs (primary consumers) to other microorganisms in the soil food web [9, 10].

Microbial interactions in complex communities, including the methanotroph interactome, have been explored using a co-occurrence network analysis based on specific genes (e.g., 16S rRNA, 18S rRNA genes) amplified from isolated nucleic acids (DNA, RNA) [11–17]. Microbial taxa that are positively correlated in the network analysis can be interpreted as having complementary roles, sharing the same habitat niche, or are driven by cross-feeding [1, 3, 11, 13, 18], whereas negative correlations are attributable to competing taxa, predation, or niche partitioning [4, 19–21]. The aerobic methanotrophs thrive in the presence of other organisms, forming (mutually) beneficial associations (e.g., receiving essential vitamins; [22]), as well as adverse relationships (e.g., selective predation by protists; [23]) with their biotic environment. These interactions can be species-specific [5, 22, 24], underscoring the relevance of the physiology, and ecological traits inherent to diverse methanotrophs in selecting for interacting partners, influencing the membership of the methanotroph interactome. Accordingly, the aerobic methanotrophs belong to Gamma- / Alpha-proteobacteria and Verrucomicrobia, with the active verrucomicrobial methanotrophs typically detected in acidic and geothermal environments (e.g., peatlands, volcanic and geothermal soils; [8, 25, 26]). These methanotrophs can be distinguished based on their physiology, including C-assimilation pathway and substrate utilization (e.g., facultative methanotrophy) and PLFA profile, among other distinct ecological characteristics [27–31]. In terrestrial ecosystems, the aerobic methanotrophs play a crucial role as a methane-biofilter at oxic-anoxic interfaces where they consume a large portion of methane produced before being emitted into the atmosphere [32], in addition to being a methane sink in well-aerated soils [33–35]. Besides the methanotrophs and interaction with methylotrophs [36, 37], very little is known of the organization (over space and time), and other constituents of the methanotroph interactome despite their relevance in modulating community function.

Here, we elaborate on the methane-driven interaction network in naturally-occurring complex communities from widespread methane hotspots (pristine/restored ombrotrophic peatlands, and paddy, riparian, and landfill cover soils). Considering that a high substrate (methane) availability favors gammaproteobacterial methanotrophs (e.g., *Methylobacter*, *Methylosarcina*; [32, 38, 39]), in turn influence the recruitment of the non-methanotrophs, we hypothesize that members of the methanotroph interactome from these environments would converge, having more shared than site-specific co-occurring taxa. To address our hypothesis, we applied stable isotope probing (SIP) using ^13^C-CH_4_ coupled to a co-occurrence network analysis of the ^13^C-enriched 16S rRNA gene, which not only enabled direct association of methanotrophic activity to the network structure, but also provided a tangible link between the co-occurring taxa involved in the trophic interaction. This is in contrast to previous work deriving the networks from isolated nucleic acids (DNA and RNA), where relationships between taxa were inferred rather than demonstrated. Capitalizing on the SIP-network analysis, we determined different scales of organization, that is, consistency of co-occurring taxa that were nested among the metabolically active sub-population between environments (spatial scale), and over time (temporal scale) in the pristine peatland to assess the stability of the network structure during the incubation. Furthermore, comparing the ^unlabelled^C- and ^13^C-based networks, we postulate that the networks derived from the DNA isolated from the soils (i.e., ^unlabelled^C-CH_4_ incubation, without SIP) would be relatively more complex because of the inclusion of the metabolically inactive community members, non-trophic interactions, and weak or spurious correlations. Hence, we examined the applicability of our methodological approach, while shedding light on the spatial and temporal organization of the methanotroph interactome.

## Results and discussion

### Aerobic methanotrophy, and environmental variables influencing the metabolically active bacterial community composition

Methanotrophic activity was detected in all environments and was within the range expected for low-affinity methane oxidation typical in methane hotspots (Table 1; [40, 41]). In these environments, the methanotrophs serve as a methane-biofilter, consuming high concentrations of methane generated in the anoxic soil layers before releasing into the atmosphere [32, 39, 42]. Although low-affinity methanotrophs were detected, some of these methanotrophs may also consume methane at (circum-)atmospheric levels, doubling as a methane sink under low methane availability [34, 43, 44]. The methanotrophic activity was corroborated by the significant increase (*p* < 0.05) in the *pmoA* gene abundance and/or the *pmoA*:16S rRNA gene abundance ratio (%) during the incubation (Table 1, Additional file 11: Table S1, Additional file 2: Figure S1), indicating methanotrophic growth.

**Table 1:** Selected soil physico-chemical properties, and methane uptake rates from methane hotspots

Environment	Location (coordinates)	Sampling time	pH	EC	Total C	Total N	NH_4_^+^	NO_3_^−^	SO_4_^2−^	CH_4_ uptake rate	References
				(mS cm^−1^)	(mg g_dw_^−1^)		(µmol g_dw_^−1^)			(µmol g_dw_^−1^ h^−1^)	
Paddy soil	Italian Rice Research Institute, Vercelli, Italy (45° 20’N, 8° 25’E)	May 2015	6.6 ± 0.05a	BD	13.9 ± 0.5a	1.3 ± 0.04a	1 ± 0.02ac	0.6 ± 0.01abc	0.8 ± 0.2a	0.44 ± 0.19b	[9]
Landfill cover	AHA Landfill, Kohlenfeld, Germany (52°22'N 9°26'E)	Jan 2020	8.81 ± 0.11b	0.07 ± 0.01a	136 ± 12b	10.1 ± 0.5bc	12.2 ± 8.3c	1.9 ± 1.2bc	28 ± 32bd	0.67 ± 0.24ab	This study
Pristine peatland	Zielony Mechacz, Poland (53°54´24´´N, 19°41´41´´E)	May 2019	4.39 ± 0.19c	BD	457 ± 4.2c	6.9 ± 1.2ab	0.2 ± 0.08b	0.6 ± 0.2bc	3.3 ± 0.6bcd	1 ± 0.26a	[8]; this study
Restored peatland	Rucianka, Poland (54°15´34´´N, 19°44´0.4´´E)	May 2019	4.68 ± 0.12c	BD	492 ± 6d	12.5 ± 0.4c	0.7 ± 0.3a	0.7 ± 0.6abc	2.3 ± 0.3c	1 ± 0.16a	[8]
Riparian soil	River Leine, Hannover, Germany (52°22′43.7"N 9°42′11.4"E)	May 2020	8.22 ± 0.18d	0.06 ± 0.01a	32.2 ± 8.4e	2.5 ± 0.8ab	0.4 ± 0.2a	0.07 ± 0.07a	1.8 ± 0.4a	0.15 ± 0.05c	This study

Importantly, assimilation of methane-derived ^13^C into the methanotrophs was evidenced by the detection of the ^13^C-DNA following density gradient fractionation in the SIP approach, which showed well-separated ^unlabelled^C- (“light”) and ^13^C-DNA (“heavy”) fractions (Additional file 3: Figures S2 & Additional file 4: Figure S3). The microorganisms derived from the ^13^C-enriched 16S rRNA gene thus represent the metabolically active, ^13^C-methane derived consuming, and replicating community members. Despite the relatively low proportion of methanotrophs (Additional file 2: Figures S1, Additional file 5: Figure S4 & Additional file 6: Figure S5), the bacterial community composition, as determined from the amplicon sequence analysis of the 16S rRNA gene in the “light” and “heavy” fractions were discernible, clearly separated along the axes in the Principal Component Analysis (PCA, Additional file 7: Figure S6 & Additional file 8: Figure S7), supporting the density gradient fractionation. However, with a relatively lower proportion of methanotrophs in the riparian soil (Additional file 2: Figure S1), differences in the “light” and “heavy” fractions were no longer reflected in the total bacterial population (i.e., at the 16S rRNA gene level; Additional file 7: Figure S6). Generally, the SIP approach not only confirmed the assimilation of ^13^C-methane by the methanotrophs, but also captured the subsequent dispersal of the ^13^C into the methane-driven soil food web.

**Fig. 1:**
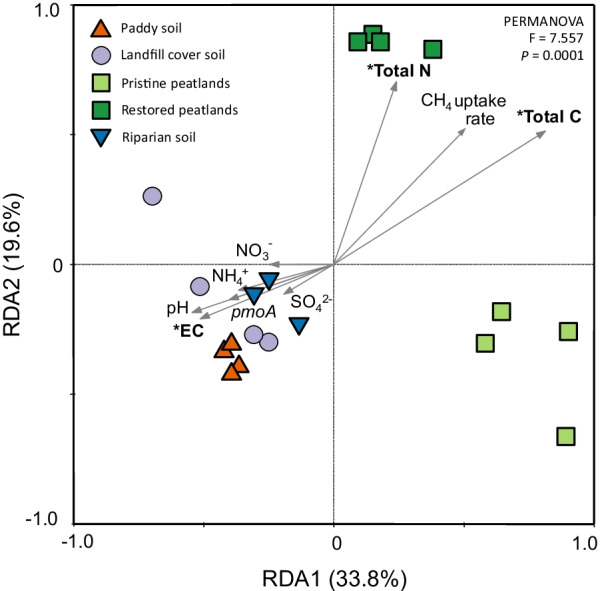
Redundancy analysis (RDA) showing compositional differences of the metabolically active bacterial community (^13^C-enriched 16S rRNA gene diversity) from widespread methane hotspots, and the variables (inorganic N, sulphate, pH, EC, total N and C, methane uptake rates, and *pmoA* gene abundance) affecting the community as constraints. Significant (*p* < 0.01) variables affecting the community composition are emboldened (EC, total C and N). Abbreviations: EC, electrical conductivity; *pmoA*, *pmoA* gene abundance as proxy for methanotroph abundance

Compositional changes during the incubation may reflect on the temporal dynamics of the bacterial, including the methanotrophic community (e.g., [45–47]). Nevertheless, with the exception of the peat, the metabolically active, that is, ^13^C assimilating and replicating bacterial community composition after the incubation was representative of the community in the starting material (Additional file 5: Figure S4). The metabolically active bacterial community composition was distinct in the ombrotrophic peatlands, as revealed in a redundancy analysis (RDA; Fig. 1). The RDA integrates the abiotic parameters in Table 1 to the ^13^C-labelled bacterial community composition in all environments. The bacterial composition in the riparian, landfill cover, and paddy soils were more similar clustering closely together, and could be separated from the community in the ombrotrophic peatlands along RDA axis 1; > 53% of the variation of the bacterial community composition could be explained by RDA 1 and RDA 2 (Fig. 1). The bacterial community composition can be profoundly influenced by the soil physico-chemical parameters including substrate availability and land use, with the latter potentially having a stronger impact on the compositional differences among the methanotrophs [9, 31, 48, 49]. Among the environmental parameters, total C and N, and electrical conductivity (EC) indicative of soil salinity, significantly (*p* < 0.05) affected the active bacterial community (Fig. 1). While EC favours the community in the riparian, landfill cover, and paddy soils, total C and N strongly affected the community particularly in the restored ombrotrophic peatland. This is not entirely unexpected as ombrotrophic peatlands are nutrient-impoverished environments, where the peat-inhabiting microorganisms would more strongly respond to C and N than in the other relatively nutrient-rich environments (Table 1; [50]). In the other environments, it is noteworthy that despite the different ecosystems represented, that is, freshwater wetlands (paddy and riparian soil) and well-aerated landfill cover soil, the active bacterial community composition was more similar, possibly forming interaction networks comprising of shared community members.

### The methanotrophic interactome over space and time

The interaction among members of the methane-driven food web was explored using a co-occurrence network analysis derived from the ^13^C-enriched 16S rRNA genes. A comparison of the networks from the different environments revealed that the ^13^C-labelled riparian soil community was relatively more connected and complex, as indicated by the higher number of interacting community members (nodes), number of connections (edges), and number of connections per node or node connectivity (average degree), but was less modular, having fewer compartmentalized groups of interaction within the network than the other environments (Table 2, Additional file 9: Figure S8; [13, 51, 52]). In contrast, the restored peatland harboured the least connected and complex interaction network (Table 2). Presumably, increased co-occurrence is fueled by a higher metabolic exchange and/or competition among members of the methanotrophic interactome in the riparian soil [18, 53].

**Table 2:** Correlations and topological properties of the co-occurrence network analysis derived from widespread methane hotspots. The networks are given in the Additional file 9: Figure S8

Network properties	Paddy soil		Landfill cover		Pristine peatland		Restored peatland		Riparian soil	
	^13^C	^Unlabelled^C	^13^C	^Unlabelled^C	^13^C	^Unlabelled^C	^13^C	^Unlabelled^C	^13^C	^Unlabelled^C
Number of nodes^a^	299	536	329	655	344	622	258	681	737	667
Number of edges^b^	980	2839	2078	8899	1684	4219	846	6918	10,003	6261
Positive edges^c^	805 (82%)	2149 (76%)	1341 (64%)	5242 (59%)	1026 (61%)	2740 (65%)	558 (66%)	4269 (62%)	7631 (76%)	4785 (76%)
Negative edges^d^	175 (18%)	690 (24%)	737 (36%)	3657 (41%)	658 (39%)	1479 (35%)	288 (34%)	2649 (38%)	2372 (24%)	1476 (24%)
Met/Met	78 (8%)	1 (0.3%)	18 (1%)	49 (0.5%)	3 (0.2%)	8 (0.5%)	19 (2.2%)	8 (0.1%)	970 (9.5%)	6 (0.1%)
Met/non-Met	318 (32%)	78 (2.7%)	365 (17%)	188 (2.5%)	177 (10.5%)	250 (5.5%)	189 (22.4%)	441 (6.9%)	3707 (37%)	331 (4.9%)
Non-Met/non-Met	584 (60%)	2760 (97%)	1695 (82%)	8662 (97%)	1504 (89.3%)	3961 (94%)	638 (75.4%)	6469 (93%)	5326 (53.5%)	5924 (95%)
Modularity^e^	0.817	1.166	1.557	2.067	2.316	1.734	1.812	1.894	0.692	0.992
Number of communities^f^	55	91	32	30	39	67	38	40	19	74
Network diameter^g^	16	14	10	10	11	10	11	11	12	10
Average path length^h^	5.913	5.803	3.969	3.607	4.402	4.372	5.01	4.228	4.201	4.433
Average degree^i^	6.55	10.59	12.63	27.173	9.79	13.566	6.55	20.317	27.14	18.774
Av. clustering coefficient^j^	0.365	0.416	0.409	0.418	0.413	0.397	0.397	0.413	0.319	0.345

*Met/Met* correlation within methanotrophs, *Met/non-Met* correlation between methanotrophs and non-methanotrophs, *Non-met/non-Met* correlation between non-methanotrphs

^a^Microbial taxon (at genus level) with at least one significant (*p* < 0.01) and strong (SparCC > 0.8 or < −0.8) correlation;

^b^Number of connections/correlations obtained by SparCC analysis;

^c^SparCC positive correlation (> 0.7 with *P* < 0.01);

^d^SparCC negative correlation (< -0.7 with *P* < 0.01);

^e^The capability of the nodes to form highly connected communities, that is, a structure with high density of between nodes connections (inferred by Gephi);

^f^A community is defined as a group of nodes densely connected internally (Gephi);

^g^The longest distance between nodes in the network, measured in number of edges (Gephi);

^h^Average network distance between all pair of nodes or the average length off all edges in the network (Gephi);

^i^The average number of connections per node in the network, that is, the node connectivity (Gephi);

^j^How nodes are embedded in their neighborhood and the degree to which they tend to cluster together (Gephi)

Because temporal community patterns may lead to the elimination of highly connected taxa [54] which affects the network complexity [13], the interaction network in the pristine peatland was additionally determined after 8, 13, and 19 days incubation to monitor the changes of the network topology over time (Table 3, Additional file 10: Figure S9). Besides being a source of methane-derived organic C, methanotrophs also drive the N-cycle by fixing N_2_ to assimilable N forms, and hence, are a key microbial group linking C and N cycling in ombrotrophic peatlands [55, 56]. The connectedness and complexity of the ^13^C-enriched 16S rRNA gene-derived interaction network, as deduced from the number of nodes, edges, and degree, fluctuated within a relatively narrow range over time when compared to the differences in the network topology between environments (Tables 2, 3). However, modularity decreased from day 8 to 13, and remained relatively unchanged thereafter, indicating a reduced number of independently connected groups of nodes or compartments within the network over time [51]. Initially, compartments that are formed centered around the methanotrophs before ^13^C dispersal to other community members at higher trophic levels. With continuous methane availability during the incubation, it is not unreasonable to assume that the methane-derived ^13^C would be more evenly and widely dispersed in the methanotrophic interactome, becoming less modular over time [9]. Such temporal changes in the network topology are anticipated given that the soil is a dynamic environment. Nevertheless, it appears that some network topological features (e.g., degree, number of nodes and edges) were relatively more consistent that others (e.g., modularity) over time.

**Table 3:** Correlations and topological properties of the co-occurrence network analysis from the pristine peatland over time. The networks are given in the Additional file 10: Figure S9

Network properties	8 days		13 days		19 days	
	^13^C	^Unlabelled^C	^13^C	^Unlabelled^C	^13^C	^Unlabelled^C
Number of nodes^a^	297	608	347	628	205	578
Number of edges^b^	1265	4651	1758	3845	687	4117
Positive edges^c^	724 (57%)	2970 (64%)	1056 (60%)	2646 (69%)	418 (61%)	2561 (62%)
Negative edges^d^	541 (43%)	1681 (36%)	702 (40%)	1199 (31%)	269 (39%)	1556 (38%)
Met/Met	4 (0.3%)	4 (0.1%)	5 (0.3%)	3 (0.1%)	8 (1.2%)	1 (0.02%)
Met/non-Met	130 (10.2%)	231 (5%)	185 (10.5%)	230 (6%)	135 (19.6%)	138 (3.3%)
Non-Met/non-Met	1131 (89.5%)	4416 (94.9%)	1568 (89.2%)	3612 (93.9%)	544 (79.2%)	3978 (96.6%)
Modularity^e^	3.317	1.752	2.440	1.445	2.478	2.150
Number of communities^f^	34	66	37	79	35	44
Network diameter^g^	14	14	11	10	17	12
Average path length^h^	5.233	4.243	4.463	4.441	5.038	4.181
Average degree^i^	8.519	15.29	10.13	12.24	6.702	14.24
Av. clustering coefficient^j^	0.421	0.404	0.415	0.399	0.430	0.374

Description of the network properties are as given in Table 2

*Met/Met* correlation within methanotrophs, *Met/non-Met* correlation between methanotrophs and non-methanotrophs, *Non-met/non-Met* correlation between non-methanotrphs

### Insights into intra-methanotroph and methanotroph/non-methanotroph interaction within the methanotrophic interactome

**Fig. 2:**
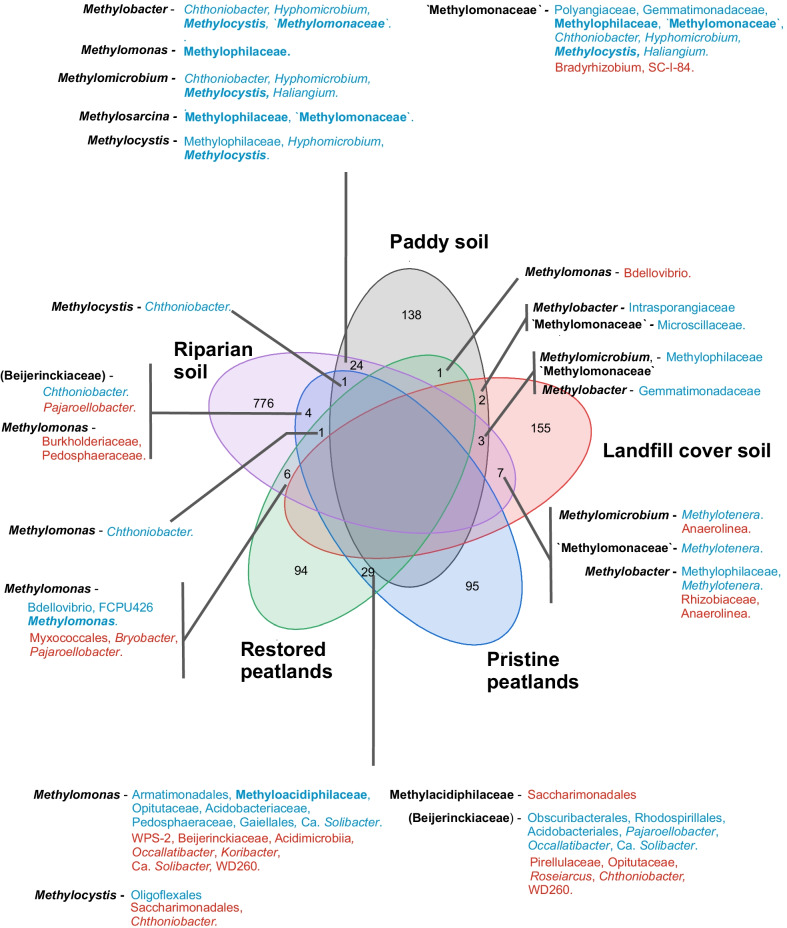
Venn diagram showing shared co-occurring taxa in all environments. Methanotrophs are emboldened. Taxa in blue and red denote significant positive and negative correlations, respectively. Beijerinkiaceae is given in brackets as many methanotrophs, along with other methylotrophs belong to this family, but remain ambiguous at the resolvable taxonomic affiliation; hence Beijerinkiaceae are potentially methanotrophs. Bacterial affiliations are identified to the highest taxonomic resolution (genus/species) whenever possible. The unique co-occurring taxa specific to each environment and classified OTUs are given in the Additional file 12: Table S2

The co-occurring methanotroph/methanotroph (intra-methanotroph) and methanotroph/non-methanotroph interactions were further explored to determine whether co-occurring taxa are conserved across different environments, and to identify non-methanotrophs as interacting partners of the methanotrophs. The non-methanotrophs and methanotrophs that co-occur are anticipated to form close associations, forming tight-knit clusters that are centered around the methanotrophs [10, 15]. On the other hand, linkages between non-methanotrophs that occurred at higher proportion (Tables 2, 3) represent heterotrophic microorganisms that assimilated the ^13^C at higher trophic levels. The co-occurring methanotroph/methanotroph and methanotroph/non-methanotroph taxa exhibited site specificity, with the majority of the co-occurring microorganisms unique to an environment (Fig. 2). Differing from our hypothesis, this suggests that microbial communities distinctly co-evolved in the different environments, and other factors besides high methane availability drives the co-occurrence of these microorganisms. Interestingly, more shared co-occurring taxa from the pristine and restored peatlands (acidic freshwater ecosystem), as well as in the riparian and paddy soil (circum-neutral freshwater ecosystems) were detected (Fig. 2), suggesting some commonalities in the environmental selection of these co-occurring microorganisms.

Communal metabolism drives the interaction network of the ^13^C-enriched members of the methanotrophic interactome [8–10, 36, 57]. Although the incorporation of ^13^C derived from dead microbial biomass can not be completely excluded, this would have been minimized with a metabolically active and growing methane-oxidizing population. Also, methane-derived ^13^C-CO_2_ may be incorporated by chemoautotrophs in the community, but the majority of the co-occurring genus/species were heterotrophs. Here, we focused on the shared taxa from the different environments which represent the more universal co-occurring members of the interaction network (Fig. 2). Expectedly, many methanotrophs (e.g., *Methylobacter*, *Methylomonas*, *Methylomicrobium*, *Methylosarcina*, *Methylocystis*, and members of Methyloacidiphilaceae) co-occur, sharing similar niche in diverse environments. Among other co-occurring taxa common to many environments, non-methanotrophic methylotrophs (e.g., *Methylotenera*, *Hyphomicrobium*, and members of Methylophilaceae) were significantly co-enriched alongside methanotrophs (Fig. 2; [8, 9, 57–59]). Indeed, cross-feeding drives their co-occurrence via passive release of methanol by the methanotrophs. Also, the non-methanotrophic methylotrophs have been demonstrated to induce the release of methanol as a C source for growth by modifying the expression of the methanol dehydrogenase in methanotrophs [36], in addition to utilizing other methane-derived one C compounds (e.g., formaldehyde, formate). Considering that some members of Myxococcales (e.g., Haliangiaceae; [60]) are recognized microbial predators shown to exert a regulatory effect in bacterial communities [61–63], their significant positive and negative correlations in the restored peatland and riparian soil suggest selective predation on the methanotrophs, as shown before in freshwater environments [64]. The apparently contrasting correlations may be explained by the predator–prey relationship, where predator and prey alternately fluctuate over time. Thus, correlations of Myxococcales with the methanotrophs may vary from positive (e.g., during nutrient availability derived from lysed cells after predation) to negative (e.g., during predation on methanotrophs) through time. Overall, although co-occurrence patterns may differ across environments, few relationships were persistent reflecting on the biological interactions that were independent of the environmental conditions. Besides predation and methylotrophic interaction, other interacting members of the methanotroph interactome remain elusive.

**Fig. 3:**
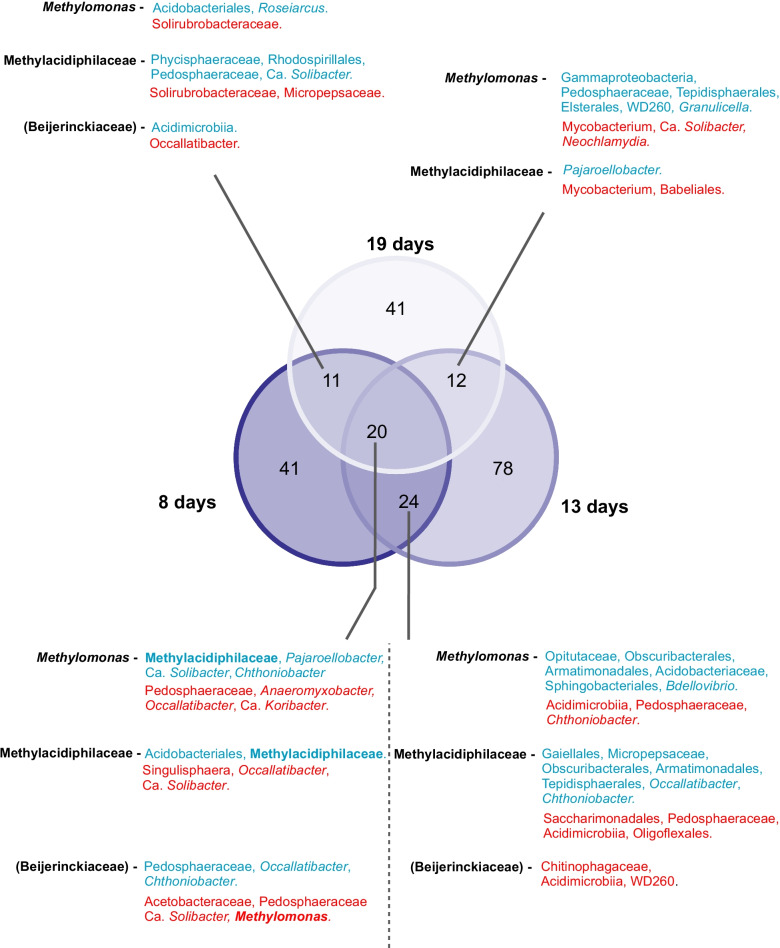
Venn diagram showing shared co-occurring taxa over time in the pristine peatland. The taxa that co-occurred at all time intervals were regarded as the “core” community members. Methanotrophs are emboldened. Taxa in blue and red denote significant positive and negative correlation, respectively. Like Fig. 2, Beijerinkiaceae is given in brackets. Bacterial affiliations are identified to the highest taxonomic resolution (genus/species) whenever possible. The unique co-occurring taxa at each time interval, and shared co-occurring taxa at two time intervals, along with the classified OTUs are given in the Additional file 13: Table S3

Of interest, *Chthoniobacter* appears to be closely associated with the methanotrophs in diverse environments (peatlands, paddy, and riparian soils), and was overwhelmingly (the only exception occurred at days 8–13 interval in the pristine peatland; Fig. 3) positively correlated to the gammaproteobacterial methanotrophs (*Methylobacter*, *Methylomicrobium*, *Methylomonas*, and other “Methylomonaceae”); *Chthoniobacter* positively and negatively correlated to the alphaproteobacterial methanotroph *Methylocystis*, depending on the environment (Fig. 2, Additional file 12: Table S2). Unlike the methylotrophs, a cultured representative of *Chthoniobacter* (*C. flavus*) is a soil-inhabiting heterotroph that cannot utilize products of methane oxidation (i.e., methanol, formate) nor other organic acids (except pyruvate) and amino acids for growth [65]. This suggests that leaked pyruvate and/or sugars derived from the ribulose monophosphate (RuMP) pathway during C-assimilation specifically in gammaproteobacterial methanotrophs may shape the cross-feeding between *Chthoniobacter* and the methanotrophs. Another co-enriched non-methanotroph taxon belonged to *Haliangium*, detected only in the paddy and riparian soils (Fig. 2, Additional file 12: Table S2). Cultured representatives of *Haliangium* (group Myxobacteria) seemingly inhabit and show a preference for mineral soils [60], corroborating with their absence in the pristine and restored peatlands (Figs. 2 and 3). However, the co-occurrence network analysis revealed statistical relationships; the biological interdependencies or causative mechanisms driving the inferred interaction requires further investigation, facilitated by co-culture studies [36, 66]. Also noteworthy is that a taxon may simultaneously be positively and negatively correlated to the same methanotroph (e.g., Ca. *Solibacter*, *Pajaroellobacter*, *Occallatibacter*; Fig. 2). Admittedly, our sequencing analysis suffers from the lack of finer taxonomic resolution. This could partly explain the seemingly contradictory correlations, which may also stem from the inherently different ecological traits possessed by members of the same genus or even strain and/or that the same microorganism may have evolved to play distinct roles in different environments [67, 68]. Hence, further exploration of the inherent microbial traits driving the co-occurrence of the methanotrophs and specific non-methanotrophs warrants attention.

Additionally, we monitored shifts in the co-occurring taxa over time (8, 13, and 19 days intervals) in the pristine peatland to determine the persistent non-methanotrophic interacting partners (Fig. 3, Additional file 13: Table S3). Unique co-occurring taxa emerged at different time intervals, with some co-occurring microorganisms overlapping between time intervals. The 20 co-occurring taxa that were consistently present at all time intervals were regarded as the core community members. The core community was thus likely to comprise microorganisms that were in close and stable interaction with the methanotrophs. Likewise, *Chthoniobacter* was consistently positively correlated to the gammaproteobacterial methanotrophs in the core community. Generally, our analysis revealed some consistency in the co-occurring patterns of the interaction network over space and time, paving the way for future detailed studies to elucidate the underlying mechanisms and metabolites driving the co-occurrence of specific taxa.

### Comparison of the interaction networks derived from the total (^unlabelled^C-DNA) and metabolically active (^13^C-DNA) microbial communities

Network analyses are commonly derived from nucleic acids isolated from the environment. Depending on the sampling strategy, the environmental samples are often collected apart and composited prior to nucleic acid extraction. Considering that microorganisms are largely restricted in their movements and particularly for the methanotrophs, strongly adhere to soil particles [69, 70], the interactions between microorganisms in these networks are thus inferred. Also, the complexity of these networks may have been overestimated given that the inferred interaction includes a large fraction of soil micoorganisms that may not be metabolically active [71, 72]. Here, we addressed these limitations by coupling ^13^C-CH_4_ SIP to a co-occurrence network analysis which provides a strong link, tracking trophic interactions of microorganisms involved in the flow of the ^13^C in the methane-based food web. Although a relatively more complex interaction topology is intuitively anticipated in networks derived from the total community (^unlabelled^C-DNA), this assumption has yet to be empirically validated. Indeed, the network structure derived from the 16S rRNA gene sequences, representing the total community exhibited higher connectivity and complexity, as indicated by the higher number of nodes, edges, and degree when compared to the network of the active community (i.e., ^13^C-enriched 16S rRNA gene sequences; Tables 2, 3). This was documented in all environments and over time in the pristine peat, except for the riparian soil where the network derived from the ^13^C-enriched 16S rRNA gene sequences was comparably more connected and complex. Hence, results largely support our postulation that the coupling of SIP to network analysis can be applied to exclude or reduce spurious connections in the networks.

The unexpected trend in the riparian soil may have been caused by methodological artifacts, namely cross-contamination of the ‘light’ (^unlabelled^C-DNA) and ‘heavy’ (^13^C-DNA) fractions, but the densities of these fractions were well-separated (Additional file 3: Figure S2). Notably, the riparian soil harboured a higher number of nodes derived from the ^13^C-enriched 16S rRNA gene (Table 2) compared to the total community (^unlabelled^C-DNA), as well as in other environments. This is indicative of a higher number of interacting microorganisms within the methanotrophic interactome in the riparian soil, which in turn, may foster higher metabolic exchange, contributing to the complexity of the methane-driven interaction network [3, 18, 73]. Whether this is the rule for networks harbouring highly diverse nodes or an exception for the riparian soil, needs further confirmation. Regardless, we demonstrate that our approach (SIP-network analysis) is an effective tool to probe trophic interactions in complex communities approximating *in-situ* conditions.

## Conclusion

Given that biological interactions modulate different aspects of microbial life in the environment, shaping the activity, biodiversity, community composition, abundance, and stability of microbial communities [8, 9, 13, 73, 74], elucidating the interaction of assembled communities within the methane-driven network is key to determining their response to environmental cues. While numerous studies utilized artificially assembled communities, we explored microbial interactions in naturally-occurring complex communities aided by SIP coupled to a co-occurrence network analysis to target the methanotroph interactome. Although co-occurring taxa were predominantly site-specific, it appears that some biological interactions (e.g., cross-feeding within methylotrophs) were independent of the environment. Results also indicate a relatively stable interaction network in the short-term, comparing networks between all environments and within the pristine peatland, with the emergence of a persistent core methanotroph interactome over time. More generally, we provide a methodological strategy to improve the network analysis derived from environmental samples by introducing SIP with labelled substrates to strengthen and substantiate the biological linkages between the potentially interacting microorganisms.

## Materials and methods

### Chemicals and reagents

Reagents (analytical and molecular biology grade) used were obtained from Carl Roth GmbH (Karlsruhe, Germany), VWR International (Hannover, Germany), and Merck (Bielefeld, Germany) unless explicitly stated otherwise. Gases (^13^C- and ^unlabelled^C-CH_4_) were ordered from Linde plc (Pullach, Germany). For ultracentrifugation, tubes, rotors, and ultra-centrifuge were sourced from Beckman Coulter (CA, USA). Further details on kits and reagents are given in the corresponding sections.

### Soil microcosm incubation, and soil physico-chemical characterization

The soils were sampled from methane-emitting environments, including a landfill cover, pristine ombrotrophic peatlands, and riparian soil (Table 1). Additionally, results from previous incubations with a rice paddy soil and ombrotrophic peatlands, were also re-analysed and included in this study [8, 9]. These environments are anticipated to harbor aerobic low-affinity methane-oxidizers. The soils were collected from the upper 10–15 cm using a corer. Three to four soil cores were collected and composited from each of four random plots spaced > 4 m apart, representing independent replicates. Samples from the peatlands (Poland; Table 1) were transported to the laboratory in ice with styrofoam containers, while the other samples (landfill cover and riparian soil; Lower Saxony, Germany) were immediately transported to the lab for incubation set-up. Because of the large amounts of waste debris, the landfill cover soil was further loosely sieved (< 5 mm) prior to incubation. Rice paddy soil was processed as described before (air-dried at room temperature and sieved to < 2 mm; [9]). The site location, sampling time, and selected soil physico-chemical properties are provided in Table 1.

The landfill cover, pristine and restored ombrotrophic peatland, and riparian soils were incubated similarly; each microcosm consisted of 5–7 g fresh soil in a 120 ml bottle. After sealing the bottle with a butyl rubber stopper crimped with a metal cap, headspace methane was adjusted to 1–2% _v/v_ (^unlabelled^C-CH_4_ and ^13^C-CH_4_, n = 4 each) in air, reflecting on the anticipated *in-situ* methane concentrations in the methane hotpots. Incubation was performed at 27 °C, while shaking (110 rpm) in the dark. Upon methane depletion, the microcosm was aerated for 30 min before replenishing headspace methane (1–2% _v/v_), and incubation resumed as before. The incubation was terminated when approximately 30 µmol CH_4_ per g fresh weight soil was consumed to ensure sufficient labelling. Furthermore, incubations were performed with samples from the pristine ombrotrophic peatland in this study to follow the temporal dynamics of the methanotrophic interactome over a 19-day incubation after approximately 14 (day 8), 30 (day 13), and 60 (day 19) µmol CH_4_ per g fresh weight peat were consumed. The incubation containing the rice paddy soil was performed differently. Here, each microcosm consisted of 10 g air-dried rice paddy soil saturated with 4.5 mL autoclaved deionized water in a Petri dish. Incubation was performed statically at 25 °C in a flux chamber after adjusting headspace methane to 1–2% _v/v_ (^unlabelled^C-CH_4_, n = 2; ^13^C-CH_4_, n = 4) in air, as detailed in [9]; incubation was terminated when approximately 30 µmol CH_4_ per g soil was consumed. In all microcosms, the soil was homogenized, sampled, and stored in the -20 °C freezer till DNA extraction after the incubation.

Methane was measured daily during the incubation using a gas chromatograph (7890B GC System, Agilent Technologies, Santa Clara, USA) coupled to a pulsed discharge helium ionization detector (PD-HID), with helium as the carrier gas. Cumulative methane uptake is reported. Inorganic nitrogen (ammonium and nitrate) concentrations were determined in autoclaved deionized water (1:1 or 1:2 _w/v_) after centrifugation and filtration (0.22 µm) with standard colorimetric methods [75, 76], while total sulphate was determined using a modified colorimetric assay after Wolfson [77]; all colorimetric assays were performed using an Infinite M plex plate reader (TECAN, Meannedorf, Switzerland). Total C and N were determined from air-dried (50 °C) and milled soils using a Vario EL III elemental analyzer (Elementar Analysensysteme GmbH, Langenselbold, Germany).

### DNA-SIP with ^13^C-CH_4_

DNA was extracted using the DNeasy PowerSoil Kit (Qiagen, Hilden, Germany) according to the manufacturer's instructions. DNA was extracted in duplicate per sample to obtain sufficient amounts for the isopycnic ultracentrifugation.

The DNA-SIP with ^13^C-CH_4_ was performed as described before [9, 78]. Isopycnic ultracentrifugation was performed using an Optima L-80XP (Beckman Coulter Inc., USA) at 144,000 g for 67 h. Immediately after centrifugation, fractionation was performed using a peristaltic pump (Duelabo, Dusseldorf, Germany) at 2.8 rpm min^−1^. Nine or ten fractions were obtained per sample, after discarding the final fraction. Fractionation was unsuccessful for one out of the four replicates of the riparian soil. The density gradient of each fraction was determined using an AR200 digital refractometer (Reichert Technologies, Munich, Germany). Thereafter, the DNA from each fraction was precipitated and washed twice with ethanol, and the pellet was re-suspended in 30 µL ultrapure PCR water (INVITROGEN, Waltham, USA). The *pmoA* gene was quantified from the precipitated DNA for each fraction using quantitative PCR, qPCR (MTOT assay; [79]) to distinguish the “heavy” (^13^C-enriched DNA) and “light” (^unlabelled^C-DNA) fractions after comparing the fractions derived from the ^13^C- and ^unlabelled^C-CH_4_ incubations (Additional file 3: Figure S2 & Additional file 4: Figure S3). The “heavy” and “light” DNA fractions were identified as defined in Neufeld et al. [78]. The 16S rRNA gene from these fractions was subsequently amplified for Illumina MiSeq sequencing and network construction.

### Quantitative PCR (qPCR)

The qPCR assay was performed to enumerate the *pmoA* gene abundance after fractionation (DNA-SIP), and to follow the change in the *pmoA* relative to the 16S rRNA gene abundance during the incubation. The increase in the *pmoA*:16S rRNA gene abundance ratio is indicative of methanotrophic growth [8], complementing the DNA-SIP. The qPCR was performed using a BIORAD CFX Connect RT System (Biorad, Hercules, USA). Each qPCR reaction (total volume, 20 µL) targeting the *pmoA* gene consisted of 10 µL SYBR 2X Sensifast (BIOLINE, London, UK), 3.5 µL of A189f/mb661r primer each (4 µM), 1 µL BSA (1%), and 2 µL template DNA. Each qPCR reaction (total volume, 20 µL) targeting the 16S rRNA gene consisted of 10 µL SYBR 2X Sensifast, 1.2 µL MgCl_2_ (50 mM), 2.0 µL of 341F/907R primer each (10 µM), 1.8 µL of PCR-grade water, 1 µL BSA (1%), and 2 µL template DNA. The PCR thermal profiles are given elsewhere [8, 79]. Template DNA was undiluted when quantifying the *pmoA* gene after fractionation, and diluted 50 or 100-fold with RNase- and DNase-free water when enumerating the *pmoA* and 16S rRNA gene from the DNA isolated from the soil. These dilutions resulted in the optimal gene copy numbers. The calibration curve, ranging from 10^1^ to 10^7^ copy number of target genes, was derived from clones (*pmoA* gene) or plasmid DNA (16S rRNA gene) as described before [47]. The PCR efficiency was on average 90–95%, depending on the qPCR assay. Amplicon specificity was assessed from the melt curve, and further confirmed by 1% agarose gel electrophoresis.

### 16S rRNA gene amplicon preparation and Illumina MiSeq sequencing

The 16S rRNA gene was amplified with the primer pair 341F/805R. Each PCR reaction (total volume, 40 µL) consisted of 20 µL KAPA HIFI (Roche, Basel, Switzerland), 2 μL forward/reverse tagged-primer each (10 μM), 2 µL BSA (1%), 4 μL template DNA, and 10 μL PCR-grade water. The template DNA was replaced with equivalent amounts of PCR-grade water and DNA derived from *Rhodanobacter denitrificans* in the negative and positive control, respectively. The positive control was confirmed after sequencing, resulting in the retrieval of sequences affiliated to *R. denitrificans*, as expected; there was no amplification in the negative control. The PCR thermal profile consisted of an initial denaturation step at 95 °C for 3 min, followed by 30 cycles of denaturation at 98 °C for 20 s, annealing at 53 °C for 15 s, and elongation at 72 °C for 15 s. The final elongation step was at 72 °C for 1 min. Amplicon specificity was verified by 1% agarose gel electrophoresis. Thereafter, the PCR product was purified using the GeneRead Size Selection Kit (Qiagen, Hilden, Germany) to be used as template (5 μL) for the second PCR. The second PCR was performed to attach the adapters to the amplicons using the Nextera XT index kit (Illumina, San Diego, USA). The reagents, reagent concentrations, and thermal profile for the second PCR are given elsewhere [9]. After the second PCR, the amplicons were purified using the MagSi-NGS^PREP^ Plus Magnetic beads (Steinbrenner Laborsysteme GmbH, Wiesenbach, Germany) according to the manufacturer’s instructions. Equimolar amounts (133 ng) of the amplicons from each sample were pooled for library preparation and sequencing using the Illumina MiSeq version 3 chemistry (paired-end, 600 cycles).

### 16S rRNA gene amplicon analyses

Firstly, the 16S rRNA gene paired-end reads were merged using PEAR [80], and subsequently processed using QIIME 2 version 2019.10. The de-multiplex and quality control steps were performed with DADA2 [81] using the consensus method to remove remaining chimeric and low-quality sequences. After filtering, approximately 5,650,000 high quality sequences were obtained, with an average of ~ 49,570 sequences per sample. Singletons and doubletons were removed, and the samples were rarefied to 11,600 sequences following the sample with the lowest number of sequences. Classification was performed at 97% similarity based on the Silva database v. 132 [82]. Because the aerobic methanotrophs are restricted to < 30 genera from two phyla [30], they were identified using the “search” function in the OTU table. The composition of the active bacterial community from different environments was visualized as a RDA based on the relative abundance of the 16S rRNA gene diversity. The data matrix was initially analysed using the detrended correspondence analysis (DCA), indicating a linear data distribution and the best-fit mathematical model was the RDA. Also, plot clustering was performed using permutational multivariate analysis of variance (PERMANOVA; [83]) to test whether the different environments harboured significantly different active bacterial communities and whether the communities in the “heavy” and “light” fractions were distinct. The PERMANOVA was calculated using PAST 4 software [84]. The RDA analysis was implemented in Canoco 4.5 (Biometrics, Wageningen, The Netherlands). The 16S rRNA gene sequences (sample names/treatments and corresponding accession numbers are listed in Additional file 14: Table S4) were deposited at the National Center for Biotechnology Information (NCBI) under the BioProject ID number PRJNA751592.

### Co-occurrence network analysis

The complexity of the interaction was explored using a co-occurrence network analysis, based on the 16S rRNA gene (OTU level) derived from the ^13^C-enriched DNA (“heavy” fraction), representing the active community. Moreover, networks were also constructed from the unlabelled DNA from the ^unlabelled^C-CH_4_ incubations to be compared to the networks derived from the ^13^C-enriched DNA. The networks were derived from at least 3 replicates. Previously, we showed that networks derived from an uneven number of replicates (e.g., 3–5) and a randomly chosen subset of replicates showed comparable results [9]. To remove weak and spurious correlations, only the OTUs with ≥ 10 sequences were included in the analysis, which represented > 90% of the total amount of sequences. The co-occurrence analysis between absolute OTUs counts were calculated using the Python module “SparCC”, a tool designed to generate and assess the correlations of the compositional data [85]. True SparCC correlations with a magnitude of > 0.8 (positive correlation) or < − 0.8 (negative correlation), and statistical significance of *p* < 0.01 were selected for the network construction. The *p*-values were obtained by 99 permutations of random selections of the data tables. All networks were constructed in parallel using the same analytical pipeline, including re-analysis of networks from the rice paddy soil and peatland together with the current dataset. This enables direct comparison of the networks derived from the different environments and over time. Assessment of the networks was based on their topological properties, which includes the number of nodes and edges, modularity, number of communities, network diameter, average path length, degree, and clustering coefficient (interpretation of these network properties are provided in Table 2; [13, 86, 87]). Additionally, the correlations between the methanotrophs, and methanotrophs/non-methanotrophs were identified to determine potential intra-methanotroph and non-methanotroph interacting partners. The network construction and topological properties were calculated with Gephi [88].

### Statistical analysis

Statistical analysis was performed in PAST 4 software [84]. Normal distribution was tested using the Shapiro–Wilk test, and homogeneity of variance was tested using Levene’s test. Where normality and homogeneity of data were met, an ANOVA with Tukey post-hoc test (*p* < 0.05) was performed for comparisons between sites and over time in the pristine peatland. Otherwise, a Kruskall-Wallis ANOVA and Dunn’s post-hoc test (*p* < 0.05) were performed.

## Supplementary Information

**Additional file 1**.** Text file**. Supplementary table and figure legends.

**Additional file 2**.** Figure S1**. The* pmoA* and 16S rRNA gene abundances in the starting material and after incubation in diverse environments (mean ± s.d.; n ≥ 4). The qPCR assay was performed in duplicate for each DNA extraction. The 16S rRNA and* pmoA* gene abundances for all samples were at least an order of magnitude higher than the lower detection limit of the qPCR assays. The upper and lower case letters indicate the level of significance (p < 0.05) of the 16S rRNA gene and* pmoA* gene abundance between environments in the starting material. The asterisk indicates significant difference (p < 0.05) in the starting* pmoA* gene abundance and after incubation. The numbers at the top of each bar refer to the pmoA:16S rRNA gene abundance ratio in percentage (%), which increased after incubation.

**Additional file 3**.** Figure S2**. Relative pmoA gene abundance along the density gradient of the ^13^C- and ^unlabelled^C-CH_4_ incubations with the (a) paddy soil, (b) landfill cover soil, (c) restored peatland, (d) pristine peatland, and (e) riparian soil (mean ± s.d.; n=4 each). The results of the paddy soil (a; [2]) and the peatlands (c,d; [1]) were re-analysed for the present study. The pmoA gene relative abundance was calculated as the proportion of each fraction over the total sum of all fractions per sample. The density gradients of the ^13^C- and ^unlabelled^C-CH4 incubations were compared to distinguish the “light” from the “heavy” fraction in the ^13^C-CH_4_ incubation. The arrows denote the “light” and “heavy” fractions where the 16S rRNA gene was amplified for Illumina MiSeq sequencing in the ^13^C-CH_4_ incubations.

**Additional file 4**.** Figure S3**. Relative* pmoA* gene abundance along the density gradient of the ^13^C- and ^unlabelled^C-CH_4_ incubations in the pristine peat at days 8, 13, and 19 (mean ± s.d.; n=4 each). The* pmoA* gene relative abundance was calculated as the proportion of each fraction over the total sum of all fractions per sample. The arrows denote the “light” and “heavy” fractions where the 16S rRNA gene was amplified for Illumina MiSeq sequencing in the ^13^C-CH_4_ incubations.

**Additional file 5**.** Figure S4**. Mean relative abundance of the methanotroph-affiliated OTUs in the paddy soil, landfill cover soil, pristine/restored peatlands, and riparian soil based on the 16S rRNA gene sequences in the starting material and after the incubation with ^13^C-methane (“light” and “heavy” fractions). The numbers at the bottom of the bars denote the mean proportion (%) of the methanotroph-affiliated OTUs among the total 16S rRNA gene sequences. Abbreviations; S.M, starting material; L, “light” fraction; H, “heavy” fraction.

**Additional file 6**.** Figure S5**. Mean relative abundance of the methanotroph-affiliated OTUs in the pristine peatland after 8, 13, and 19 days incubation with ^13^C-methane (“light” and “heavy” fractions), based on the 16S rRNA gene sequences. The numbers at the bottom of the bars denote the mean proportion (%) of the methanotroph-affiliated OTUs among the total 16S rRNA gene sequences.

**Additional file 7**.** Figure S6**. Principal component analysis showing the clustering of the 16S rRNA gene sequences in the “light” and “heavy” fractions of the (a) paddy soil (orange, triangle), (b) landfill cover soil (purple, circle), (c) pristine peatland (light green, square), (d) restored peatland (dark green, square), and (e) riparian soil (blue, inverted triangle). All replicates (n=4) are given; in the incubation with the riparian soil, fractionation was unsuccessful for one replicate. Full colored and striped symbols represent the “light” and “heavy” fraction, respectively.

**Additional file 8**.** Figure S7**. Principal component analysis showing the clustering of the 16S rRNA gene sequences in the ‘light’ and ‘heavy’ fractions of the pristine peatland over time (days 8, 13, and 19). All replicates (n=4) are given. Full colored and striped symbols represent the ‘heavy’ and ‘light’ fraction, respectively.

**Additional file 9**.** Figure S8**. Co-occurrence network analysis of methane hotspots derived from the ^13^C- and ^unlabelled^C-DNA. The corresponding topological parameters of the networks are provided in Table 2. Each node represents a bacterial taxon at the OTU level, while the size and shade of the node corresponds to the number of connections per node and the number of connections passing through the node (i.e., darker shade for nodes acting as a bridge between other nodes at higher frequencies), respectively. A connection denotes significant SparCC correlation (p<0.01) with a magnitude of > 0.8 (positive correlation, blue edges) or < -0.8 (negative correlations, red edges).

**Additional file 10**.** Figure S9**. Co-occurrence network analysis after 8, 13, and 19 days incubation of the pristine peat derived from the ^13^C- and ^unlabelled^C-DNA. The corresponding topological parameters of the networks are provided in Table 3. Each node represents a bacterial taxon at the OTU level, while the size and shade of the node corresponds to the number of connections per node and the number of connections passing through the node (i.e., darker shade for nodes acting as a bridge between other nodes at higher frequencies), respectively. A connection denotes significant SparCC correlation (p<0.01) with a magnitude of > 0.8 (positive correlation, blue edges) or < -0.8 (negative correlations, red edges).

**Additional file 11**.** Table S1**. Selected physico-chemical parameters and methane uptake rates of individual replicates in methane hotspots (rice paddy soil, landfill cover soil, pristine peatland, restored peatland, and riparian soil). Summarized data given in** Table 1**.

**Additional file 12**.** Table S2**. Signficantly positively and negatively co-occuring (p < 0.01) OTUs between environments, as determined by the co-occurrence network analysis. The first panel shows site-specific co-occurring OTUs, while the other panels show shared co-occurring OTUs between environments. The OTUs were given to the finest resolveable taxonomic affiliation based on the Silva database v. 132, whenever available. The number in brackets refer to the OTU numbers. Abbreviations: pos, positive correlations; neg, negative correlations; RP, rice paddy; LC, landfill cover soil; PP, pristine peatland; RP, restored peatland; RS, riparian soil; MIP, methanotroph interacting partner (including other co-occurring methanotrophs).

**Additional file 13**.** Table S3**. Signficantly positively and negatively co-occuring (p < 0.01) OTUs in the pristine peatland over time (days 8, 13, and 19, respectively denoted by T1, T2, and T3), as determined by the co-occurrence network analysis. The first panel shows co-occurring OTUs at each time interval while the other panels show shared co-occurring OTUs between time intervals. The OTUs were given to the finest resolveable taxonomic affiliation based on the Silva database v. 132, whenever available. The number in brackets refer to the OTU numbers. Abbreviations: pos, positive correlations; neg, negative correlations; T1, after 8 days incubation; T2, after 13 days incubation; T3, after 19 days incubation; MIP, methanotroph interacting partner (including other co-occurring methanotrophs).

**Additional file 14**.** Table S4**. Sample names/treatment and corresponding accession numbers (BioProject PRJNA751592). Sample name is labelled in the following order: site, sampling time, 12C or 13C (i..e, ^unlabelled^C or ^13^C-CH4 incubations), H or L (i.e., “heavy” or “light” fractions). Note that for the pristine peatland, T1 and T3 correspond to days 8 and 19, respectively; samples from day 13 are published (Table 1; [1]).

## Data Availability

The sequencing data generated in this study were deposited to the National Center for Biotechnology Information (NCBI) under the BioProject ID number PRJNA751592. The sequencing data can be assessed using the following web link: https://www.ncbi.nlm.nih.gov/bioproject/PRJNA751592. All other data generated or analysed during this study were included in this article and its Additional files.

## Abbreviations

ANOVA: Analysis of variance
BSA: Bovine serum albumin
DNA: Deoxyribonucleic acid
OTU: Operational taxonomic unit
PCA: Principal component analysis
PCR: Polymerase chain reaction
PD-HID: Pulsed discharge helium ionization detector
PERMANOVA: Permutational multivariate analysis of variance
PLFA: Phospholipid fatty acids
pmoA: Particulate methane monooxygenase Subunit A
qPCR: Quantitative polymerase chain reaction
RDA: Redundancy analysis
RNA: Ribonucleic acid
SIP: Stable isotope probing
