# Supplementary material for: The methane-driven interaction network in terrestrial methane hotspots
Source: Environ Microbiome. 2022 Apr 5;17:15. doi: 10.1186/s40793-022-00409-1 (PMC8981696; doi:10.1186/s40793-022-00409-1)
Supplement: Supplementary file 1 — Additional file 1. Text file. Supplementary table and figure legends. [file 40793_2022_409_MOESM1_ESM.pdf]

Supplementary Information

The methane-driven interaction network in terrestrial methane hotspots.

Thomas Kaupper[1], Lucas W. Mendes[2], Anja Poehlein[3], Daria Frohloff[1], Stephan Rohrbach[1], Marcus A. Horn[1]*, Adrian Ho[1]*.

[1]Institute for Microbiology, Leibniz Universität Hannover, Herrenhäuser Str. 2, 30419 Hannover, Germany.

[2]Center for Nuclear Energy in Agriculture, University of São Paulo CENA-USP, Brazil.

[3]Department of Genomic and Applied Microbiology and Göttingen Genomics Laboratory, Institute of Microbiology and Genetics, George-August University Göttingen, Grisebachstr. 8, D-37077 Göttingen, Germany.

*For correspondence: Adrian Ho (adrian.ho@ifmb.uni-hannover.de), Marcus A. Horn (horn@ifmb.uni-hannover.de).

Running title: The methanotroph interactome.

Included:

- **Supplementary tables.**
- **Supplementary figures and figure captions**

Table S1: Selected physico-chemical parameters and methane uptake rates of individual replicates in methane hotspots (rice paddy soil, landfill cover soil, pristine peatland, restored peatland, and riparian soil). Summarized data given in Table 1.

Table S2: Signficantly positively and negatively co-occuring ($p < 0.01$) OTUs between environments, as determined by the co-occurrence network analysis. The first panel shows site-specific co-occurring OTUs, while the other panels show shared co-occurring OTUs between environments. The OTUs were given to the finest resolveable taxonomic affiliation based on the Silva database v. 132, whenever available. The number in brackets refer to the OTU numbers. Abbreviations: pos, positive correlations; neg, negative correlations; RP, rice paddy; LC, landfill cover soil; PP, pristine peatland; RP, restored peatland; RS, riparian soil; MIP, methanotroph interacting partner (including other co-occurring methanotrophs).

Table S3: Signficantly positively and negatively co-occuring ($p < 0.01$) OTUs in the pristine peatland over time (days 8, 13, and 19, respectively denoted by T1, T2, and T3), as determined by the co-occurrence network analysis. The first panel shows co-occurring OTUs at each time interval while the other panels show shared co-occurring OTUs between time intervals. The OTUs were given to the finest resolveable taxonomic affiliation based on the Silva database v. 132, whenever available. The number in brackets refer to the OTU numbers. Abbreviations: pos, positive correlations; neg, negative correlations; T1, after 8 days incubation; T2, after 13 days incubation; T3, after 19 days incubation; MIP, methanotroph interacting partner (including other co-occurring methanotrophs).

Table S4: Sample names/treatment and corresponding accession numbers (BioProject PRJNA751592). Sample name is labelled in the following order: site, sampling time, 12C or 13C (i..e, $^{unlabelled}C$ or ^{13}C-CH_4 incubations), H or L (i.e., “heavy” or “light” fractions). Note that for the pristine peatland, T1 and T3 correspond to days 8 and 19, respectively; samples from day 13 are published (Table 1; [1]).

Figure Legends

Figure S1: The *pmoA* and 16S rRNA gene abundances in the starting material and after incubation in diverse environments (mean ± s.d.; n≥4). The qPCR assay was performed in duplicate for each DNA extraction. The 16S rRNA and *pmoA* gene abundances for all samples were at least an order of magnitude higher than the lower detection limit of the qPCR assays. The upper and lower case letters indicate the level of significance ($p<0.05$) of the 16S rRNA gene and *pmoA* gene abundance between environments in the starting material. The asterisk indicates significant difference ($p<0.05$) in the starting *pmoA* gene abundance and after incubation. The numbers at the top of each bar refer to the *pmoA*:16S rRNA gene abundance ratio in percentage (%), which increased after incubation.

Figure S2: Relative *pmoA* gene abundance along the density gradient of the ^{13}C- and $^{unlabelled}C$-CH_4 incubations with the (a) paddy soil, (b) landfill cover soil, (c) restored peatland, (d) pristine peatland, and (e) riparian soil (mean ± s.d.; *n*=4 each). The results of the paddy soil (a; [2]) and the peatlands (c,d; [1]) were re-analysed for the present study. The *pmoA* gene relative abundance was calculated as the proportion of each fraction over the total sum of all fractions per sample. The density gradients of the ^{13}C- and $^{unlabelled}C$-CH_4 incubations were compared to distinguish the “light” from the “heavy” fraction in the ^{13}C-CH_4 incubation. The arrows denote the “light” and “heavy” fractions where the 16S rRNA gene was amplified for Illumina MiSeq sequencing in the ^{13}C-CH_4 incubations.

Figure S3: Relative *pmoA* gene abundance along the density gradient of the ^{13}C- and $^{unlabelled}C$-CH_4 incubations in the pristine peat at days 8, 13, and 19 (mean ± s.d.; *n*=4 each). The *pmoA*

gene relative abundance was calculated as the proportion of each fraction over the total sum of all fractions per sample. The arrows denote the “light” and “heavy” fractions where the 16S rRNA gene was amplified for Illumina MiSeq sequencing in the ^{13}C-CH_4 incubations.

Figure S4: Mean relative abundance of the methanotroph-affiliated OTUs in the paddy soil, landfill cover soil, pristine/restored peatlands, and riparian soil based on the 16S rRNA gene sequences in the starting material and after the incubation with ^{13}C-methane (“light” and “heavy” fractions). The numbers at the bottom of the bars denote the mean proportion (%) of the methanotroph-affiliated OTUs among the total 16S rRNA gene sequences. Abbreviations; S.M, starting material; L, “light” fraction; H, “heavy” fraction.

Figure S5: Mean relative abundance of the methanotroph-affiliated OTUs in the pristine peatland after 8, 13, and 19 days incubation with ^{13}C-methane (“light” and “heavy” fractions), based on the 16S rRNA gene sequences. The numbers at the bottom of the bars denote the mean proportion (%) of the methanotroph-affiliated OTUs among the total 16S rRNA gene sequences.

Figure S6: Principal component analysis showing the clustering of the 16S rRNA gene sequences in the “light” and “heavy” fractions of the (a) paddy soil (orange, triangle), (b) landfill cover soil (purple, circle), (c) pristine peatland (light green, square), (d) restored peatland (dark green, square), and (e) riparian soil (blue, inverted triangle). All replicates (n=4) are given; in the incubation with the riparian soil, fractionation was unsuccessful for one replicate. Full colored and striped symbols represent the “light” and “heavy” fraction, respectively.

Figure S7: Principal component analysis showing the clustering of the 16S rRNA gene sequences in the 'light' and 'heavy' fractions of the pristine peatland over time (days 8, 13, and 19). All replicates (n=4) are given. Full colored and striped symbols represent the 'heavy' and 'light' fraction, respectively.

Figure S8: Co-occurrence network analysis of methane hotspots derived from the ^{13}C- and $^{unlabelled}C$-DNA. The corresponding topological parameters of the networks are provided in Table 2. Each node represents a bacterial taxon at the OTU level, while the size and shade of the node corresponds to the number of connections per node and the number of connections passing through the node (i.e., darker shade for nodes acting as a bridge between other nodes at higher frequencies), respectively. A connection denotes significant SparCC correlation ($p<0.01$) with a magnitude of > 0.8 (positive correlation, blue edges) or < -0.8 (negative correlations, red edges).

Figure S9: Co-occurrence network analysis after 8, 13, and 19 days incubation of the pristine peat derived from the ^{13}C- and $^{unlabelled}C$-DNA. The corresponding topological parameters of the networks are provided in Table 3. Each node represents a bacterial taxon at the OTU level, while the size and shade of the node corresponds to the number of connections per node and the number of connections passing through the node (i.e., darker shade for nodes acting as a bridge between other nodes at higher frequencies), respectively. A connection denotes significant SparCC correlation ($p<0.01$) with a magnitude of > 0.8 (positive correlation, blue edges) or < -0.8 (negative correlations, red edges).

References

[1] Kaupper T, Mendes LW, Harnisz M, Krause SMB, Horn MA, Ho A. Recovery of methanotrophic activity is not reflected in the methane-driven interaction network after peat mining. App Environ Microbiol. 2021b; doi.org/10.1128/AEM.02355-20.

[2] Kaupper T, Mendes LW, Lee HJ, Mo Y, Poehlein A, Jia Z, Horn MA, Ho A. When the going gets tough: Emergence of a complex methane-driven interaction network during recovery from desiccation-rewetting. Soil Biol Biochem. 2021a; doi.org/10.1016/j.soilbio.2020.108109.
