# Supplementary figures and images for: The methane-driven interaction network in terrestrial methane hotspots
Source: Environ Microbiome. 2022 Apr 5;17:15. doi: 10.1186/s40793-022-00409-1 (PMC8981696; doi:10.1186/s40793-022-00409-1)

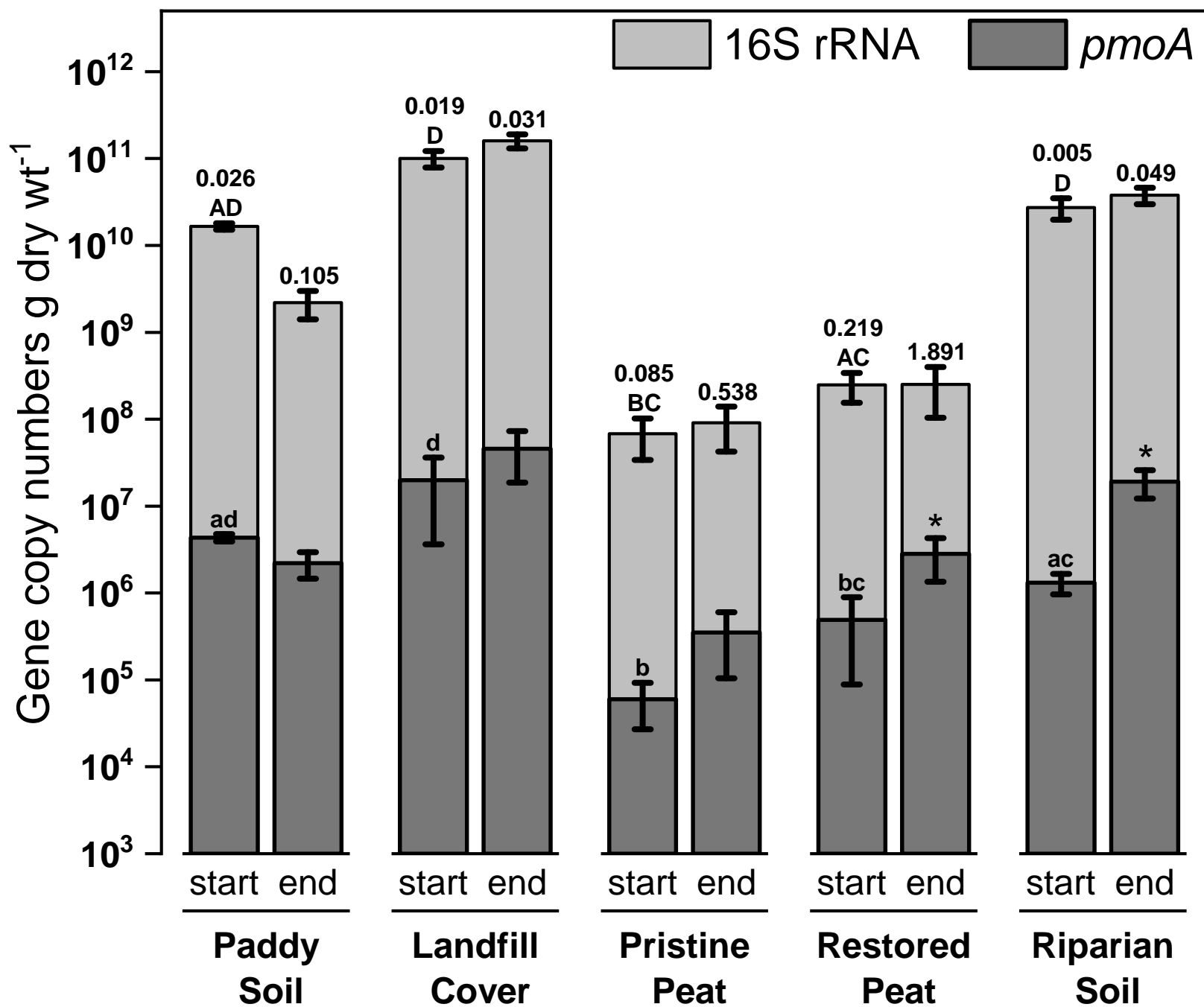

Supplement: Supplementary file 2 — Additional file 2. Figure S1. The pmoA and 16S rRNA gene abundances in the starting material and after incubation in diverse environments (mean ± s.d.; n ≥ 4). The qPCR assay was performed in duplicate for each DNA extraction. The 16S rRNA and pmoA gene abundances for all samples were at least an order of magnitude higher than the lower detection limit of the qPCR assays. The upper and lower case letters indicate the level of significance (p < 0.05) of the 16S rRNA gene and pmoA gene abundance between environments in the starting material. The asterisk indicates significant difference (p < 0.05) in the starting pmoA gene abundance and after incubation. The numbers at the top of each bar refer to the pmoA:16S rRNA gene abundance ratio in percentage (%), which increased after incubation. [file 40793_2022_409_MOESM2_ESM.pdf]

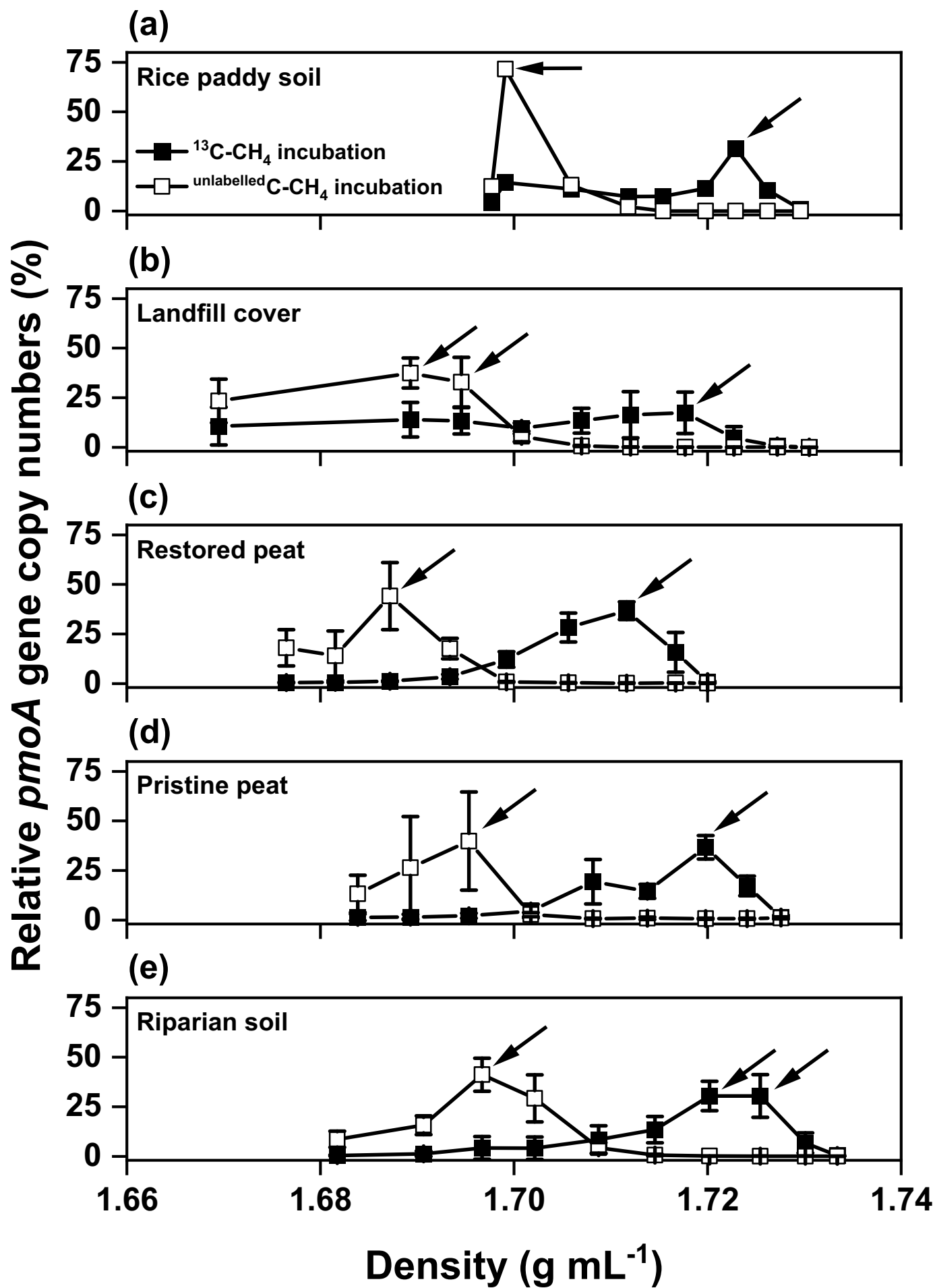

Figure S2

Supplement: Supplementary file 3 — Additional file 3. Figure S2. Relative pmoA gene abundance along the density gradient of the 13C- and unlabelledC-CH4 incubations with the (a) paddy soil, (b) landfill cover soil, (c) restored peatland, (d) pristine peatland, and (e) riparian soil (mean ± s.d.; n=4 each). The results of the paddy soil (a; [2]) and the peatlands (c,d; [1]) were re-analysed for the present study. The pmoA gene relative abundance was calculated as the proportion of each fraction over the total sum of all fractions per sample. The density gradients of the 13C- and unlabelledC-CH4 incubations were compared to distinguish the “light” from the “heavy” fraction in the 13C-CH4 incubation. The arrows denote the “light” and “heavy” fractions where the 16S rRNA gene was amplified for Illumina MiSeq sequencing in the 13C-CH4 incubations. [file 40793_2022_409_MOESM3_ESM.pdf]

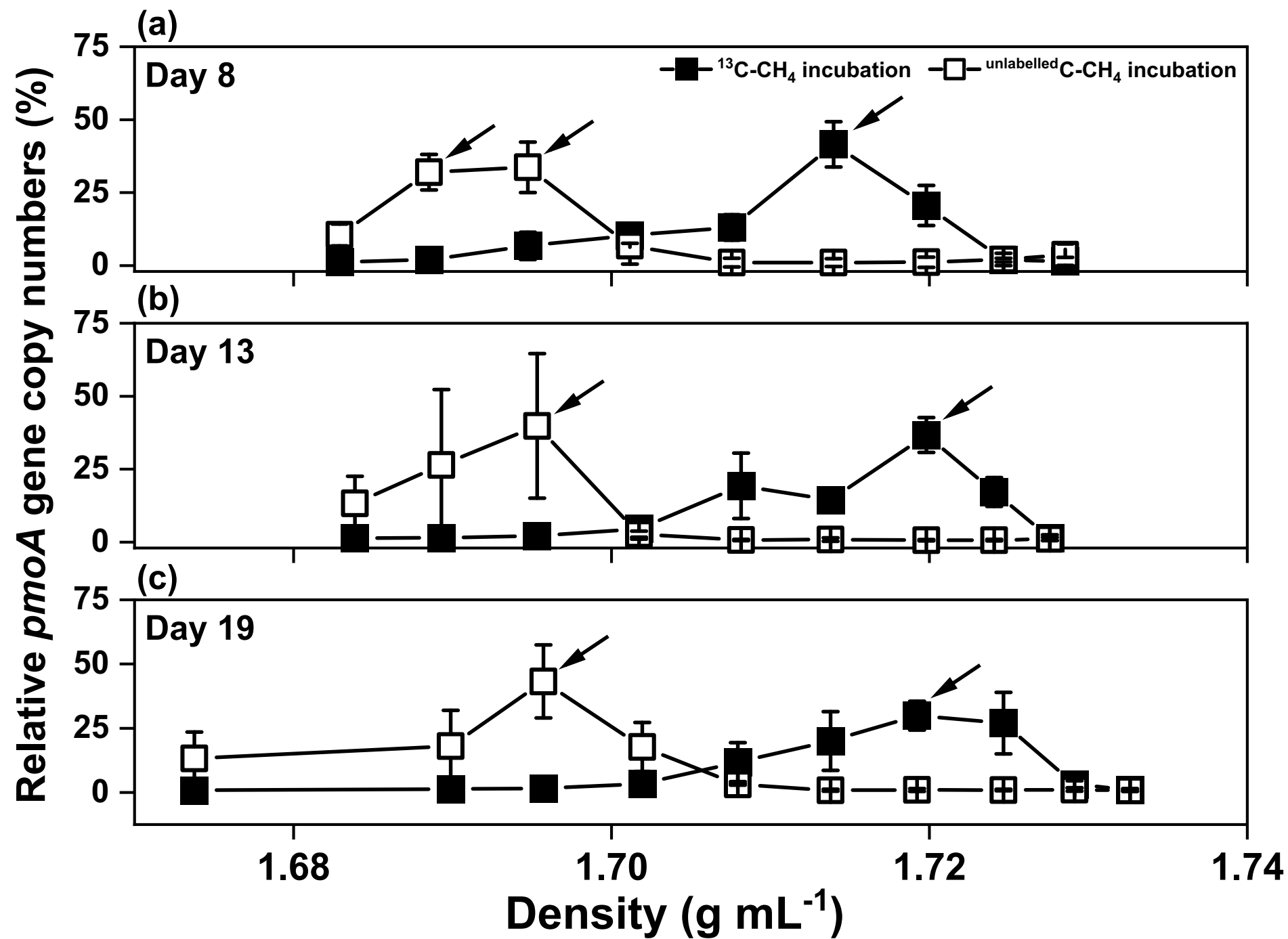

Figure S3

Supplement: Supplementary file 4 — Additional file 4. Figure S3. Relative pmoA gene abundance along the density gradient of the 13C- and unlabelledC-CH4 incubations in the pristine peat at days 8, 13, and 19 (mean ± s.d.; n=4 each). The pmoA gene relative abundance was calculated as the proportion of each fraction over the total sum of all fractions per sample. The arrows denote the “light” and “heavy” fractions where the 16S rRNA gene was amplified for Illumina MiSeq sequencing in the 13C-CH4 incubations. [file 40793_2022_409_MOESM4_ESM.pdf]

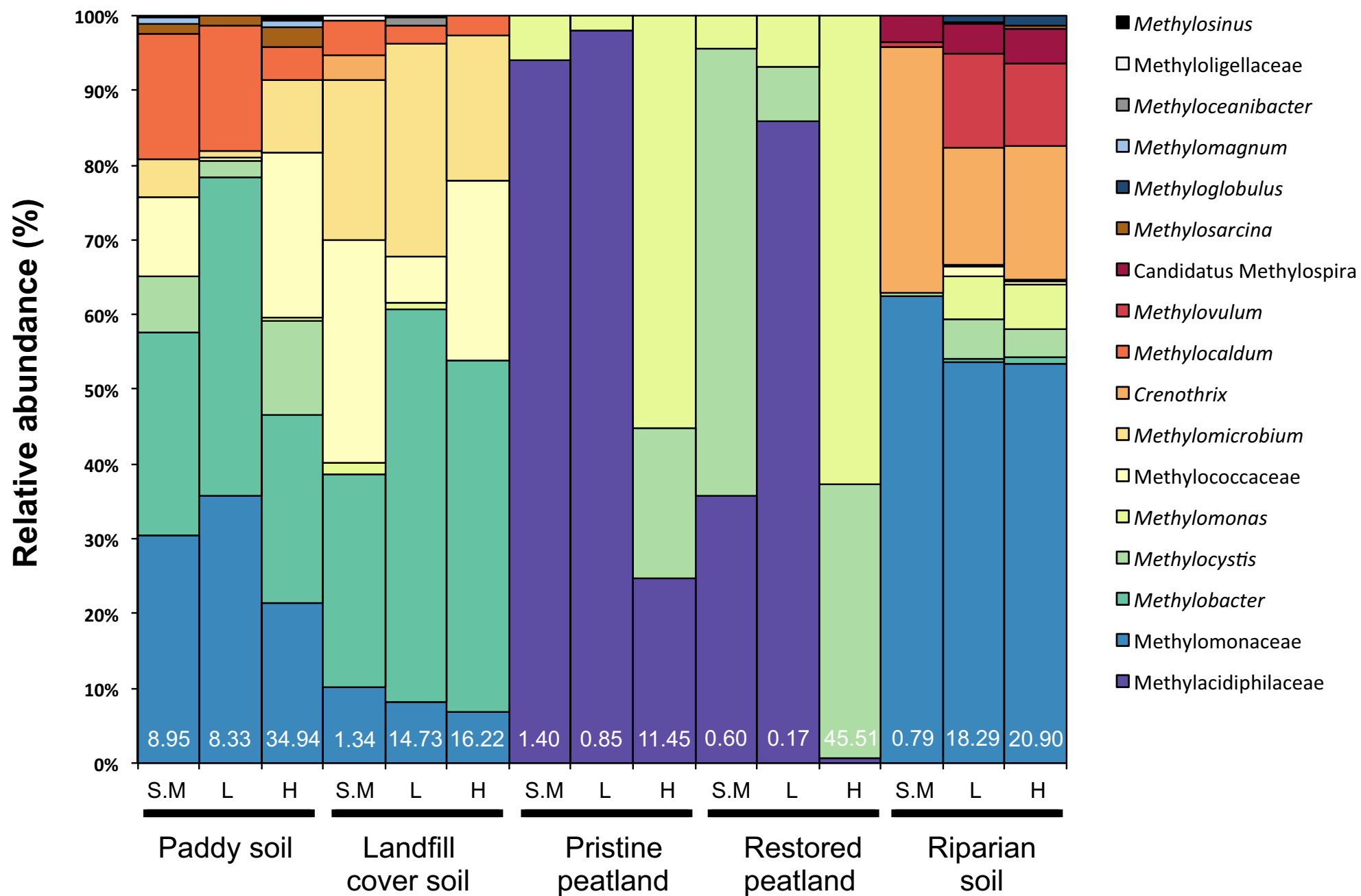

**Figure S4**

Supplement: Supplementary file 5 — Additional file 5. Figure S4. Mean relative abundance of the methanotroph-affiliated OTUs in the paddy soil, landfill cover soil, pristine/restored peatlands, and riparian soil based on the 16S rRNA gene sequences in the starting material and after the incubation with 13C-methane (“light” and “heavy” fractions). The numbers at the bottom of the bars denote the mean proportion (%) of the methanotroph-affiliated OTUs among the total 16S rRNA gene sequences. Abbreviations; S.M, starting material; L, “light” fraction; H, “heavy” fraction. [file 40793_2022_409_MOESM5_ESM.pdf]

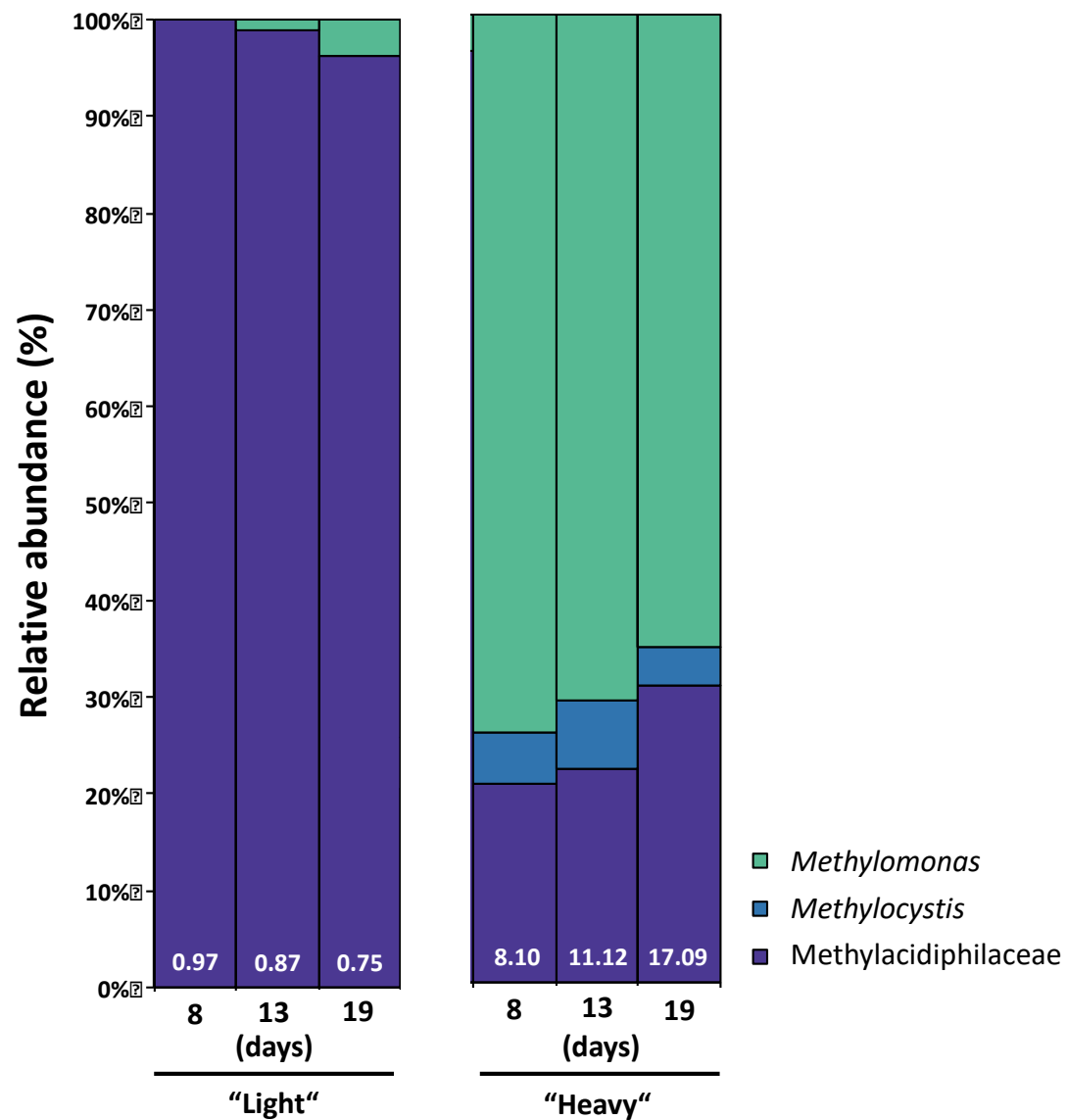

Figure S5

Supplement: Supplementary file 6 — Additional file 6. Figure S5. Mean relative abundance of the methanotroph-affiliated OTUs in the pristine peatland after 8, 13, and 19 days incubation with 13C-methane (“light” and “heavy” fractions), based on the 16S rRNA gene sequences. The numbers at the bottom of the bars denote the mean proportion (%) of the methanotroph-affiliated OTUs among the total 16S rRNA gene sequences. [file 40793_2022_409_MOESM6_ESM.pdf]

(a) Rice paddy soil

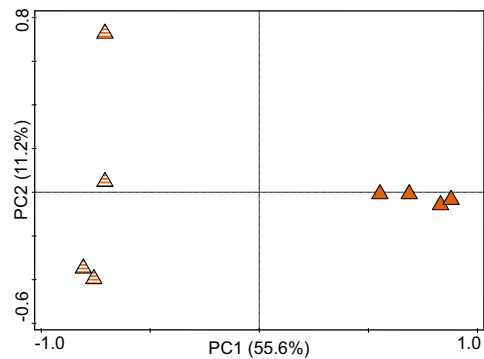

(b) Landfill cover

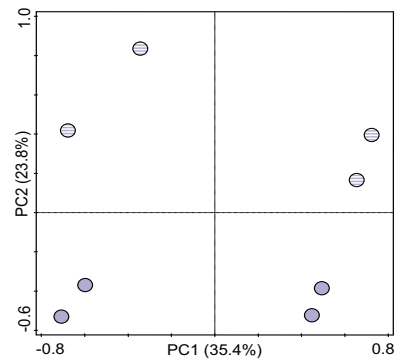

(d) Pristine peat

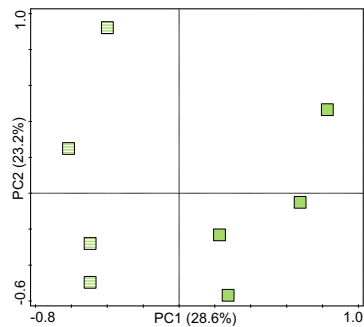

(e) Restored peat

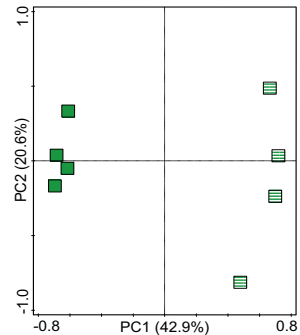

(f) Riparian soil

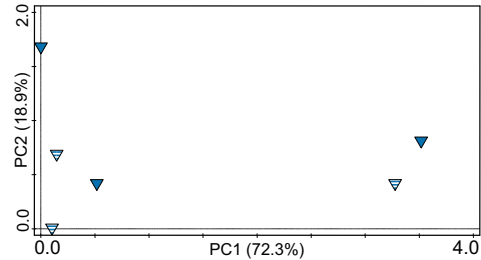

**Figure S6**

Supplement: Supplementary file 7 — Additional file 7. Figure S6. Principal component analysis showing the clustering of the 16S rRNA gene sequences in the “light” and “heavy” fractions of the (a) paddy soil (orange, triangle), (b) landfill cover soil (purple, circle), (c) pristine peatland (light green, square), (d) restored peatland (dark green, square), and (e) riparian soil (blue, inverted triangle). All replicates (n=4) are given; in the incubation with the riparian soil, fractionation was unsuccessful for one replicate. Full colored and striped symbols represent the “light” and “heavy” fraction, respectively. [file 40793_2022_409_MOESM7_ESM.pdf]

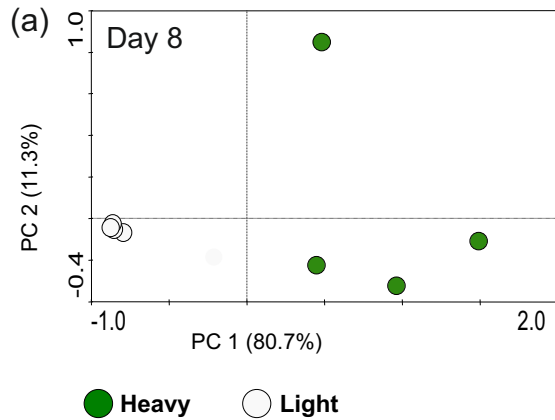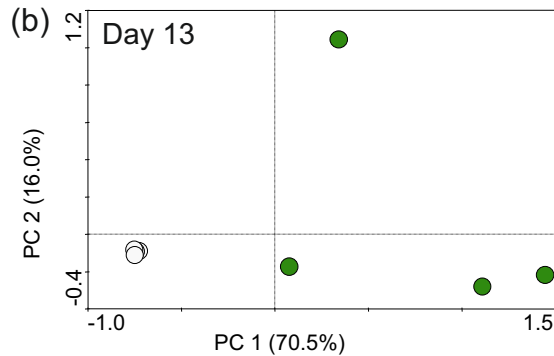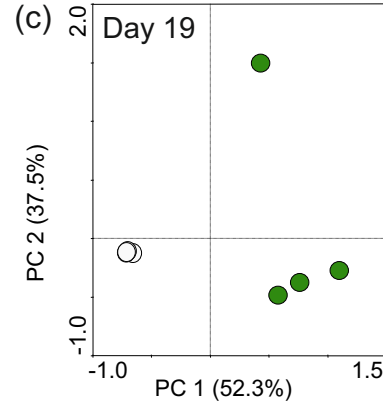

**Figure S7**

Supplement: Supplementary file 8 — Additional file 8. Figure S7. Principal component analysis showing the clustering of the 16S rRNA gene sequences in the ‘light’ and ‘heavy’ fractions of the pristine peatland over time (days 8, 13, and 19). All replicates (n=4) are given. Full colored and striped symbols represent the ‘heavy’ and ‘light’ fraction, respectively. [file 40793_2022_409_MOESM8_ESM.pdf]

Day 8

Day 13

Day 19

$^{13}\text{C}$

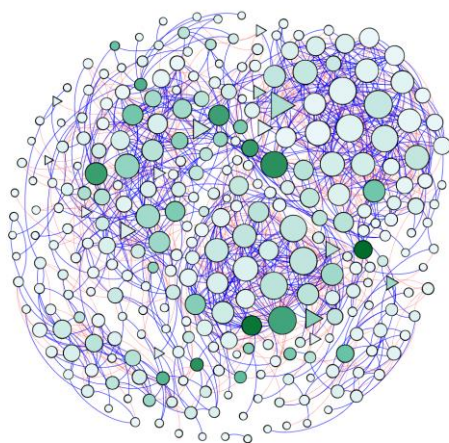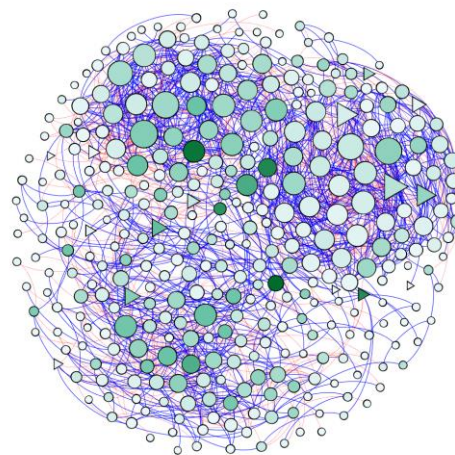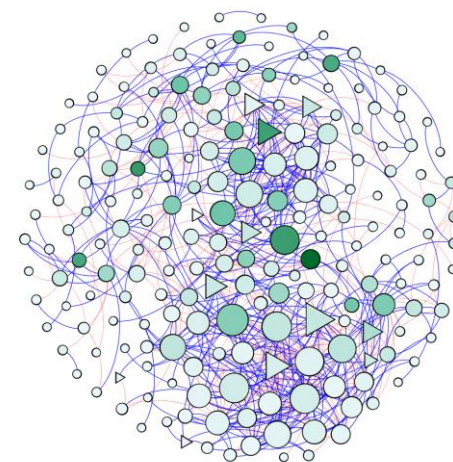

Unlabelled  $\text{C}$

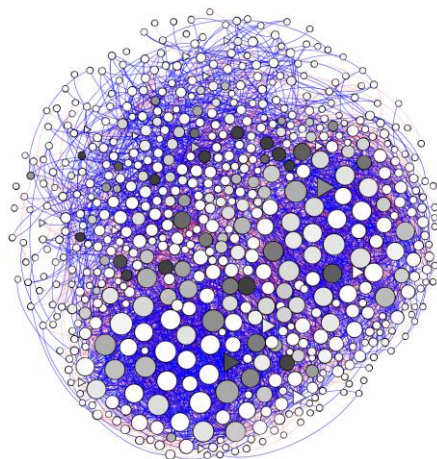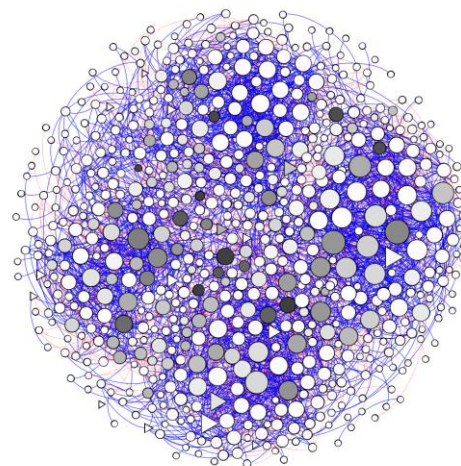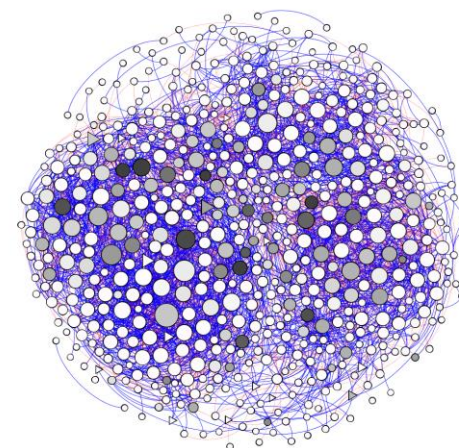

Figure S9

Supplement: Supplementary file 10 — Additional file 10. Figure S9. Co-occurrence network analysis after 8, 13, and 19 days incubation of the pristine peat derived from the 13C- and unlabelledC-DNA. The corresponding topological parameters of the networks are provided in Table 3. Each node represents a bacterial taxon at the OTU level, while the size and shade of the node corresponds to the number of connections per node and the number of connections passing through the node (i.e., darker shade for nodes acting as a bridge between other nodes at higher frequencies), respectively. A connection denotes significant SparCC correlation (p<0.01) with a magnitude of > 0.8 (positive correlation, blue edges) or < -0.8 (negative correlations, red edges). [file 40793_2022_409_MOESM10_ESM.pdf]
