# Supplementary material for: The methane-driven interaction network in terrestrial methane hotspots
Source: Environ Microbiome. 2022 Apr 5;17:15. doi: 10.1186/s40793-022-00409-1 (PMC8981696; doi:10.1186/s40793-022-00409-1)
Supplement: Supplementary file 9 — Additional file 9. Figure S8. Co-occurrence network analysis of methane hotspots derived from the 13C- and unlabelledC-DNA. The corresponding topological parameters of the networks are provided in Table 2. Each node represents a bacterial taxon at the OTU level, while the size and shade of the node corresponds to the number of connections per node and the number of connections passing through the node (i.e., darker shade for nodes acting as a bridge between other nodes at higher frequencies), respectively. A connection denotes significant SparCC correlation (p<0.01) with a magnitude of > 0.8 (positive correlation, blue edges) or < -0.8 (negative correlations, red edges). [file 40793_2022_409_MOESM9_ESM.pdf]

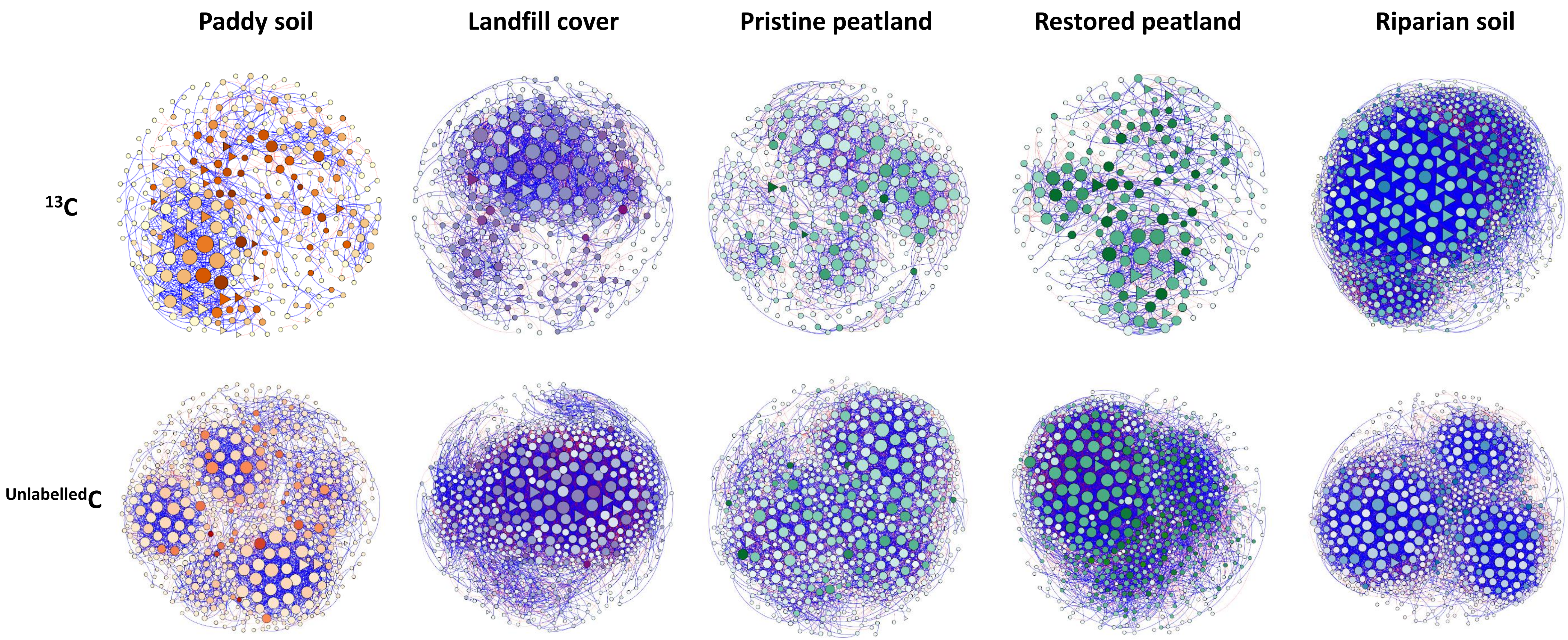
Paddy soil
Landfill cover
Pristine peatland
Restored peatland
Riparian soil
13C
Unlabelled C

Figure S8
